# Fexofenadine HCl enhances growth, biofilm, and lactic acid production of *Limosilactobacillus reuteri* and *Bifidobacterium longum*: implications for allergy treatment

**DOI:** 10.1186/s12866-025-04130-0

**Published:** 2025-07-11

**Authors:** Zainab Kamel Hammouda, Reham Wasfi, Nourtan F. Abdeltawab

**Affiliations:** 1 Department of Microbiology and Immunology, Faculty of Pharmacy, October University for Modern Sciences and Arts (MSA), Giza, Egypt; 2 Department of Microbiology and Immunology, Faculty of Pharmacy, Cairo University, Cairo, Egypt

**Keywords:** Fexofenadine HCl, *Limosilactobacillus reuteri*, *Bifidobacterium longum*, Lactic acid, Cyproheptadine HCl, Desloratadine

## Abstract

**Background:**

It is evident that various drugs influence the gut microbiota, yet the precise mechanism driving these effects remain ambiguous. Considering the growing recognition of gut microbiota’s role in health and disease, it is important to explore how commonly used drugs, such as antihistamines, may alter microbial composition and function. Histamine, an essential interkingdom signaling molecule, shapes bacterial virulence, biofilm formation, and immune regulation. However, the effects of antihistamines on bacterial colonization are mostly unknown. This study aimed to investigate the potential effects of antihistamine exposure on critical factors which affect the pathogenicity and colonization of selected gut bacterial species, such as growth, biofilm formation, and adherence to cell lines, at intestinal concentrations. If antihistamines influence bacterial metabolism or composition, they may consequently affect Short Chain Fatty Acid (SCFA) production, which could have downstream effects on gut homeostasis and immune function. Specifically, we examined the impact of three antihistamines – fexofenadine HCl, cyproheptadine HCl, and desloratadine –on bacteria from the four dominant gut phyla: *Bifidobacterium longum*,* Limosilactobacillus reuteri*,* Bacteroides fragilis*, and *Escherichia coli*.

**Results:**

Our results showed that cyproheptadine HCl and desloratadine inhibited the growth of all tested bacteria, whereas fexofenadine HCl promoted the growth of all species except B. *longum*. Furthermore, cyproheptadine HCl and desloratadine reduced the biofilm-forming capacity of these bacterial species and altered their effects on adherence to Caco-2/HT-29 cell lines aligning with changes in cell surface hydrophobicity: increased cell surface hydrophobicity correlated with greater bacterial adherence to surfaces. In contrast, fexofenadine HCl enhanced biofilm formation and adherence of *B. longum* and *L. reuterii* in Caco-2/HT-29 co-cultures. It also led to increased production of lactic and propionic acids, with a statistically significant increase observed in acetic acid levels (*p* < 0.05).

**Conclusion:**

In summary, our findings suggest that fexofenadine HCl, unlike cyproheptadine HCl and desloratadine, supports the growth, and colonization of probiotic bacteria such as *L. reuteri* and *B. longum* with potential anti allergic benefits, and enhancing their SCFA production. Conversely, cyproheptadine HCl and desloratadine suppressed bacterial growth, hinting at potential antimicrobial properties that may warrant exploration for drug repurposing.

## Introduction

The gut microbiota, also known as the gut flora, refers to the diverse community of microorganisms residing in the human gastrointestinal tract. This community comprises bacteria, viruses, fungi, and archaea, all of which play a crucial role in maintaining human health. Such role is carried out by a range of functions such as aiding in digestion, regulating the immune system, producing vitamins and metabolites, and preventing colonization by harmful pathogens [1].

The gut microbiota is typically dominated by two main phyla of bacteria, Bacteroidetes and Firmicutes, while other phyla such as Actinobacteria, Proteobacteria, and Verrucomicrobia are present in smaller proportions [2, 3]. The composition of the gut microbiota is highly dynamic and influenced by factors such as diet, lifestyle, age, genetics, and environmental exposures [2, 4–6]. Research suggests that the gut microbiota plays a role in drug metabolism. Population-based studies have revealed associations between various non-antibiotic drug classes and specific gut microbiome signatures, suggesting that these interactions may contribute to disease development. Antihistamines, which are commonly used for extended periods, have attracted research interest due to their potential effects on the gut microbiota composition [7, 8].

Bacteria have developed advanced signalling mechanisms to interact with their environment, including engagement with host-derived molecules such as neurotransmitters and biogenic amines [9]. One such molecule is histamine which play a crucial role in mediating inter-domain communication, influencing bacterial behaviour and virulence. Certain bacterial species can sense and react to histamine, altering their motility, adhesion, and gene expression [9]. For example, in the human pathogen *Pseudomonas aeruginosa*, histamine sensing initiates a chemo attractive response and regulates the expression of several virulence-associated genes [10]. Research has also demonstrated that many bacteria can synthesize and secrete histamine. In the human gut, bacterial histamine release has been shown to influence host immune responses, and at elevated levels, it can contribute to host pathologies [11]. These interactions suggest that antihistamines, by interfering with histamine signaling, may influence bacterial behavior beyond direct growth effects, which can alter gut microbiota composition and function.

There are two generations of H1 antihistamines: the first generation, such as cyproheptadine HCl, and the more selective second generation including desloratadine and fexofenadine HCl [12]. Despite growing interest, there is limited research on the direct effects of antihistamines on gut bacterial growth, biofilm formation, and short-chain fatty acids (SCFAs) production at intestinal concentrations. Given the increasing evidence of drug-microbiota interactions, understanding these effects is crucial for elucidating how antihistamines may alter the microbial balance and influence gut health, potentially impacting immune regulation and potential disease prevention. The influence of the drug on the microbiota includes changes in colonization factors such as growth, biofilm formation, and adherence to intestinal cells, which can favor the abundance or depletion of specific gut bacteria [13, 14]. Research has shown that certain Lactic Acid Bacteria (LAB) strains can regulate the balance of T-helper 1 (Th1) and T-helper 2 (Th2) cells, promoting a shift towards a Th1 response, which is associated with reduced allergic inflammation. Additionally, LAB can stimulate the production of anti-inflammatory cytokines such as IL-10 and regulatory T cells (Tregs), enhancing immune tolerance and reducing allergic symptoms. Some LAB strains have also been shown to improve epithelial barrier function, thereby reducing allergen penetration and subsequent allergic responses [15–17].

To address this gap, this study aims to examine the effects of first-generation (cyproheptadine HCl) and second-generation (fexofenadine HCl and desloratadine) antihistamines on the growth, biofilm formation, and adherence to intestinal cell lines. The selected bacteria -*Limosilactobacillus reuteri*, *Bifidobacterium longum*, *Bacteroides fragilis*, and *Escherichia coli* – represent key members of the gut microbiota. By focusing on the impact of antihistamines on these key gut bacterial species, the study seeks to provide a clearer understanding of the potential benefits or risks associated with long-term antihistamine use. This research may highlight the differential effects of different generations of antihistamines on gut microbiota, suggesting a potential role in modulating gut microbial balance with antiallergic benefits. The findings may inform future research on drug repurposing and strategies to maintain or restore gut microbiota balance in individuals using antihistamines.

## Materials and methods

### Microorganisms and growth conditions

Four bacterial strains, *Bacteroides fragilis* (DSM 2151), *Bifidobacterium longum* (DSM 20219), *Escherichia coli* (ATCC 8739), and *Limosilactobacillus reuteri* (ATCC 23272) were selected for this study as representatives for the four main bacterial phyla in the gut microbiota, Bacteroidetes, Actinobacteria, Proteobacteria, and Firmicutes, respectively.

All strains were recovered in their recommended media: *Bacteroides fragilis* was cultured on Brain-heart infusion supplemented (BHIS) broth for 48 h, and then on neomycin anaerobic blood agar (NABA) for an additional 48 h; *Bifidobacterium longum* was cultured on Reinforced Clostridial Media (RCM) broth for 24 h, and then streaked on De Man, Rogosa & Sharpe (MRS) agar supplemented with 0.05% cysteine for 48 h; Luria Bertani (LB) broth and MacConkey agar for E. coli, each for an overnight culture. Anaerobic conditions were used to incubate the previous cultures at 37ˊC. The plates/tubes were incubated in a BBL anaerobic jar, Gas pack Anaerobic system, Franklin, New Jersey, to produce anaerobiosis. After growing on selective media, bacteria were mixed with an equal volume of 50% glycerol (resulting in 25% final concentration) and stored at −20 °C in sterile Eppendorf [18].

### Preparation of antihistaminic drugs stock solutions

Three antihistamine drugs were used in this study; cyproheptadine HCl belong to the first-generation H1 antagonists, desloratadine and fexofenadine HCl belongs to the second-generation H1 antagonist. Desloratadine, cyproheptadine HCl, and fexofenadine HCl were obtained in pharmaceutical grade from EPICO (Giza, Egypt), Vasudha Pharma (Telangana, India), and India-swift Laboratories (Chandigarh, India), respectively. Each drug was prepared by dissolving drugs in the least amount of Dimethyl Sulfoxide (DMSO) and drug stocks were preserved at −20 °C. Desloratadine, cyproheptadine HCl, and fexofenadine HCl were utilized at final concentrations of 5.3619, 4.1168, and 37.165 µM, respectively, based on intestinal concentrations previously determined by Maier et al. [19].

### Evaluating the effect of antihistaminic drugs on bacterial growth

This assay was carried out according to Hammouda et al. [20]. Modified Gifu anaerobic medium (mGAM), GAM broth (Nissui Pharmaceutical (Tokyo, Japan) was used for the screening experiment, based on its ability to recapitulate the abundance of different bacterial species in the human gut microbiome [19]. A well-isolated colony from each bacterial strain was cultured in mGAM medium overnight at 37 °C under anaerobic conditions. To make sure the culture was robust and consistent, the procedure was carried out twice. The final drug concentration in each well was equivalent to their intestinal concentration. Parallel technical and biological replicates were done to monitor experiment performance and to capture any biological changes, respectively. To abolish the effect of DMSO on bacterial growth the growth of bacteria in DMSO-free medium was compared to their growth in DMSO-containing medium. The viable count of bacterial growth at zero and 24 h. was determined by the drop plate method on a selective medium [21].

### Evaluating the effect of antihistaminic drugs on bacterial auto-aggregation, cell surface hydrophobicity, and biofilm formation

It has been suggested that determining a bacteria’s auto-aggregation ability and surface hydrophobicity can serve as a measure of the organism’s adhesion capacity. The hydrophobic or hydrophilic nature of the bacterial cell surface is a crucial factor in bacterial adhesion to living and nonliving surfaces [22].

#### Bacterial cell surface hydrophobicity and auto-aggregation

The bacterial strains were cultured in 5 mL selective broth media with each of the three antihistamines at their intestinal concentrations, followed by incubation for 18 h. at 37 °C under anaerobic conditions. Positive control of bacteria grown with DMSO at the same concentration used in dissolving the drug was used. Following incubation, tubes were centrifuged for 10 min at 9500 rpm, washed with ice-cold phosphate buffer saline, and further used for carrying both auto-aggregation and cell surface hydrophobicity assays [23].

The auto-aggregation assay was carried out following Botes et al. [23], where the OD_600_ of the bacteria in the saline medium was set at 0.3. One milliliter of the adjusted bacterial suspension was centrifuged at 2000 rpm for 2 min in a sterile Eppendorf tube. The OD_600_ of the supernatant was determined both immediately (A_0_) and after 1 h (A_60_) [23, 24]. The percentage of auto-aggregation was calculated using the following equation:

$$\%\;\mathrm{Auto}-\mathrm{aggregation}\;=\;\left(\frac{(\mathrm A0\;-\;\mathrm A60)}{\mathrm A0}\right)\times100\%$$

The MATH (Microbial Adhesion to Hydrocarbons) assay was used to measure the hydrophobicity of bacterial cells with all the tested drugs. All bacteria have been evaluated for the degree of hydrophobicity on their cell surfaces using a modified version of the MATH assay as described in [20]. The bacterial OD_500_ was adjusted to 0.5. In a glass tube, 4.8 mL of each bacterial suspension was combined with 0.8 mL of xylene and vigorously shaken for 1 min. Bacterial cells were left for distribution between the organic and aqueous phases for 60 min at room temperature. The absorbance of the aqueous phase was measured at the wavelength of 500 nm using a UV-visible spectrophotometer after careful removal using a micropipette. The percentage hydrophobicity was calculated using this formula:

$$\%\;\mathrm{Hydrophobicity}\;=\;\left[1\;-\;\left(\frac{\mathrm A}{\mathrm A0}\right)\right]\times100\%$$

where A_0_ is the OD_500_ of the bacteria before mixing with the xylene and A is the OD of the aqueous phase after mixing with xylene.

### Biofilm formation

Anti-histaminic drugs’ impact on bacterial biofilm formation was examined using a crystal violet assay as described in [25, 26]. Each bacterium was incubated for 24 h. at 37 °C under anaerobic conditions on a medium supplemented with 1% glucose. *L. reuteri*,* E. coli*,* B. fragilis*, and *B. longum* were cultured in Peptone yeast glucose (PYG), LB, BHIS, and RCM, respectively. Bacterial suspension cell density was adjusted to OD_600_ = 1 and then diluted 1:100 in fresh medium. A volume of 100 µl of drugs (2x intestinal concentration) was added to 100 µl of bacterial cell suspension and was inoculated in each well of a 96-well flat bottom plate (Greiner Bio-one^®^, Germany). The plates were then incubated for 48 h. (except for *E. coli* and *B. fragilis*, which were incubated for 24 h.) under anaerobic conditions. Following incubation, bacterial growth was measured at 630 nm using a microtiter plate reader (STAT FAX 2200, Awareness Technology, Florida, USA). After decanting the bacterial culture, the wells were washed twice with PBS to remove any non-adhered bacterial cells. After the adhered cells were fixed and dried in the oven for an hour at 60 °C, they were stained for 30 min with 200 µl crystal violet (0.1%). After a gentle three-time wash in water, the plates were dried in a 60 °C oven for an hour. A mixture of alcohol: acetone (80:20) was used to solubilize the stain from Gram-positive *B. longum* and *L. reuteri* [25], whereas acetic acid (33%) was used to solubilize the stain from Gram-negative *B. fragilis* and *E. coli* [26]. The optical density (OD) was determined after transferring 150 µl of the solubilized crystal violet to a fresh 96-well plate and reading the OD at 545 nm in a microtiter plate reader [27, 28], The biofilm formation index was calculated using this formula:

$$\mathrm{Biofilm}\;\mathrm{formation}\;\mathrm{index}\;=\;\frac{OD545\;-\;ODcontrol}{OD\mathit{630}\mathit\;\mathit-\mathit\;ODcontrol}$$

In addition to the test, a positive control consisting of bacteria and DMSO was also run simultaneously. Each bacterial strain had five technical replicates and three biological replicates done to compensate for potential variability.

### Evaluating the effect of antihistaminic drugs on bacterial adherence to cell lines

#### Preparation of cell lines co-culture

Co-culture cells of Caco-2/HT29 (90:10) were used to simulate intestinal tissue which was prepared according to the method described by Kleiveland [29]. Caco-2 (ATCC HTB-37) and HT29 (ATCC HTB-29) cell lines were derived from colorectal adenocarcinoma. Roswell Park Memorial Institute (RPMI) was used for cell line culturing for tested bacteria except *E.coli* because RPMI was reported to increase the susceptibility of *Enterobacteriaceae* to antibiotics added to the culturing medium thus inhibiting bacterial growth [30], therefore the DMEM medium was used instead of RPMI for *E. coli.* The confluence of cells was regularly checked by examination under an inverted microscope.

#### Cytotoxicity assay of antihistamines on cell line

The 3, −4,5 dimethyithiazol-2,5 diphenyl tetrazolium bromide (MTT) assay was used to determine the impact of three drug concentrations (2x, 1x, and 0.5x) and DMSO on cell line viability according to [31]. Cells were seeded on a microtiter flat bottom plate using RPMI media supplemented with 2% foetal bovine serum co-culture, and they were then incubated overnight at 37 °C in 5% CO_2_. For every concentration, three technical replicates were employed. Plates were incubated for 24 h at 37 °C with 10% CO_2_. Before being incubated for two hour at 37 °C with 5% CO_2_, MTT was added to each well and dissolved in fresh medium at a concentration of 0.05%. Following incubation, the medium was taken out, and solubilization was accomplished using 100 µl of DMSO [31, 32]. The color was assessed at 545 nm, and the results were utilized for calculating the percentage of viability using this formula:

$$\%\;\mathrm{Cell}\;\mathrm{viability}\;=\;\:\frac{Average\:OD\:of\:drugs\:or\:DMSO\:treated\:cell\:lines\:coculture\:}{Average\:OD\:of\:untreated\:cell\:lines\:coculture}\times\:100\%$$

#### Adherence assay

The adherence assay was performed following previously established protocols [20, 32, 33] with some modifications. The bacterial strains were grown on their specific media for 20 h. under anaerobic conditions at 37 °C. To adjust the cell density for each bacterium to 1 × 10^8^ CFU/mL, PBS was used to wash the pellet twice after centrifuging the bacteria at 6000 rpm for 5 min at 4 °C. Except for *E. coli*, which was cultivated and kept alive in DMEM media in a 24-well flat-bottom plate, the Caco-2/HT-29 co-culture was grown and maintained using RPMI medium. A 100 µl of adjusted bacterial suspension was added to 100 µl of medications at non-cytotoxic concentrations in a microtiter plate that had been seeded with cell line co-culture. Plates were incubated in 5% CO_2_ at 37 °C for 2 h. Adhered cells were lysed by treating them with 0.1% Triton X-100 for 10 min at room temperature and the reaction was stopped by the addition of 900 µl of fresh medium. The viable bacterial cells were quantified both before incubation with the cell line and after 2 h of incubation using the drop plate technique on a selective medium [20, 33, 34]. The count was calculated, and the percentage of adherent bacterial cells was determined using the following formula:

$$\%\;\mathrm{Adhered}\;\mathrm{cells}\;=\:\frac{\text{C}\text{F}\text{U}\:\text{o}\text{f}\:\text{a}\text{d}\text{h}\text{e}\text{r}\text{e}\text{d}\:\text{c}\text{e}\text{l}\text{l}\text{s}\:\left[2\:\text{h}\right]}{CFU\:of\:initial\:inoculum\:\left[0\:h\right]}\times\:100\%$$

#### Imaging of cell adherence to cell line

The protocol for preparation of adherence assay for imaging via scanning electron microscopy (SEM) was like the previously mentioned method in Sect. 2.5.3 using 12 well flat bottom plates. Following a 2-hour incubation period, the plate underwent two washes with PBS. Subsequently, a 5% glutaraldehyde solution, prepared in 0.1 M sodium cacodylate, was introduced for a 2-hour fixation process. The dehydration of the wells was achieved through a process of sequential immersion in ethanol of varying concentrations (25%, 50%, 70%, 80%, and 90%) for a duration of 10 min. per concentration, carried out at ambient temperature. The final concentration employed for the process of dehydration was 100% and the duration of exposure was 15 min. The wells underwent a process of gold coating and were subsequently subjected to examination via a scanning electron microscope (Quanta 250 FEG, West Bengal, India), utilizing magnification level of 5000x.

### Screening the effect of antihistaminic drugs on the production of short chain fatty acids by bacteria using HPLC

We aimed at understanding the impact of antihistamine drugs on short-chain fatty acids (SCFAs) production by gut bacteria. We tested the effect of fexofenadine HCl on the production of SCFAs by two bacterial species *B. longum and L. reuteri*. These two species are known for their ability to ferment complex carbohydrates into SCFAs [20, 35]. The bacterial cultures were incubated with the intestinal concentration of fexofenadine HCl at 37 °C for 16 h. under anaerobic conditions. Following incubation, the samples were centrifuged, and the supernatant was used as the analytical sample. SCFA content in the cultures was separated by Agilent1260 infinity HPLC Series (Agilent, USA), equipped with a specific organic acid analysis column Rezex^@^ ROA-organic acid H^+^ (8%) 300 × 7.80 mm (Phenomenex, California, USA) under the same condition described by Hammouda et al. [36]. A standard curve generated with known concentrations of lactic, acetic, propionic, oxalic and butyric acids allowed for accurate SCFA quantification. For comparison, positive control included bacterial cultures grown with a solvent (DMSO) at a concentration equivalent to that present in the drug solutions. The data acquisition and integration were performed using clarity Chrom software (DataApex, Praha, Czechia) [37]. Three biological replicates of each sample were analyzed.

### Statistical analysis

Statistical analysis was performed using GraphPad Prism 9.1.1 (GraphPad Software Inc., CA, USA). Changes in bacterial growth, auto-aggregation, hydrophobicity, and biofilm formation after drug treatment were compared to drug-free samples using multiple unpaired t-tests and multiple comparisons using the Holm-sidák method. The Mann-Whitney t-test was employed for statistical analysis of the bacterial viable count in adherence assay to cell line co-culture. The statistical examination of the impact of drug dilutions on the viability of the intestinal cell lines was performed using an unpaired t-test. *p* < 0.05 was used as the threshold for statistical significance. For each bacterial species and each SCFA, differences between untreated and treated groups were evaluated by unpaired two-tailed t-tests (Welch’s correction), with p-values adjusted using the Holm-Šidák method. A threshold of *p* < 0.05 was considered statistically significant.

## Results

### Antihistamines affected the growth of bacterial strains

**Fig. 1:**
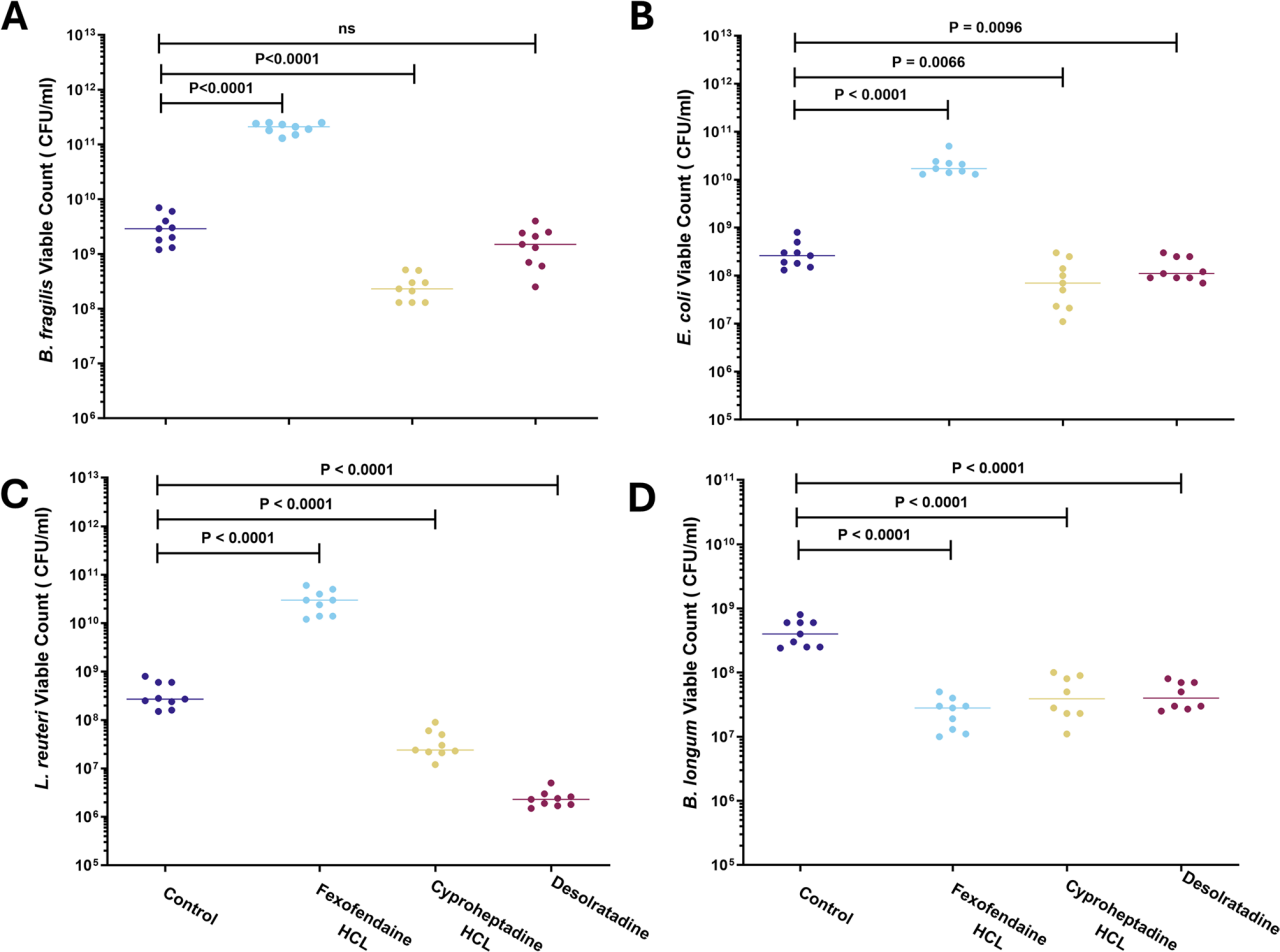
Growth of bacteria under the influence of antihistamine drugs. The effect on Gram-negative bacteria: (**A**) *Bacteroides fragilis* and (**B**) *Escherichia coli*, and Gram-positive bacteria: (**C**) *Limosilactobacillus reuteri*, and (**D**) Bifidobacterium *longum*, is represented as viable count (CFU/mL). Tested antihistamines include fexofenadine HCl, cyproheptadine HCl, and desloratadine at intestinal concentrations of 37.165 µM, 4.1168 µM, and 5.3619 µM, respectively. The control group reflects bacterial growth in the presence of DMSO. Statistical significance was assessed using the Mann-Whitney test, with “ns” indicating no significant difference

Fexofenadine HCl significantly increased (*p* < 0.0001) the viable count of *B. fragilis* (Fig. 1A), *L. reuteri* (Fig. 1C), and *E. coli* (Fig. 1B) by 2 logs. In contrast, a significant (*p* < 0.0001) reduction by 2 logs was observed in the growth of *B. longum* (Fig. 1D). Cyproheptadine HCl reduced the growth of all bacterial strains under study by 1 to 2 logs. Similarly, desloratadine reduced bacterial growth except for *B. fragilis* where its effect was insignificant.

### Effect of tested drugs on the bacterial auto-aggregation and cell surface hydrophobicity

**Fig. 2:**
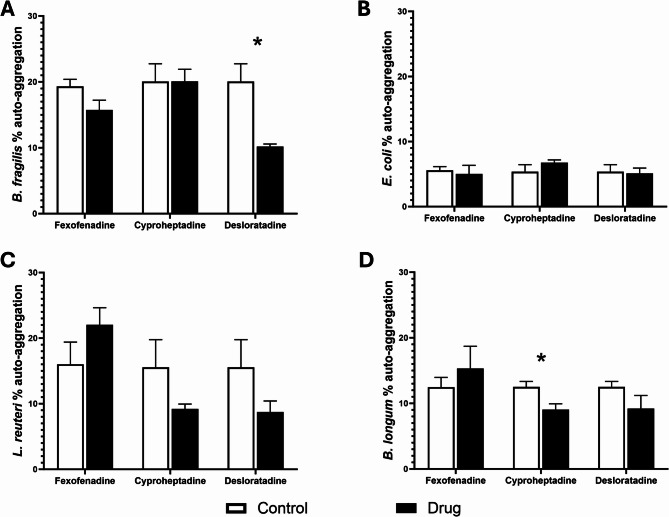
Auto-aggregation of bacteria in the presence of antihistamine drugs. Changes in the percentage of auto-aggregation were measured for (**A**) *Bacteroides fragilis*, (**B**) *Escherichia coli*, (**C)***Limosilactobacillus reuteri*, and (**D**) Bifidobacterium *longum*, after incubation with antihistamines at 37 °C for 60 min. Results are expressed as mean percentages from three independent experiments, with error bars representing standard error (SE). Tested antihistamines include fexofenadine HCl, cyproheptadine HCl, and desloratadine at intestinal concentrations of 37.165 µM, 4.1168 µM, and 5.3619 µM, respectively. The control group included bacteria with DMSO. Statistical analysis was performed using multiple unpaired t-tests with Holm-Šídák correction for multiple comparisons. * Significant difference (*p* < 0.05)

**Fig. 3:**
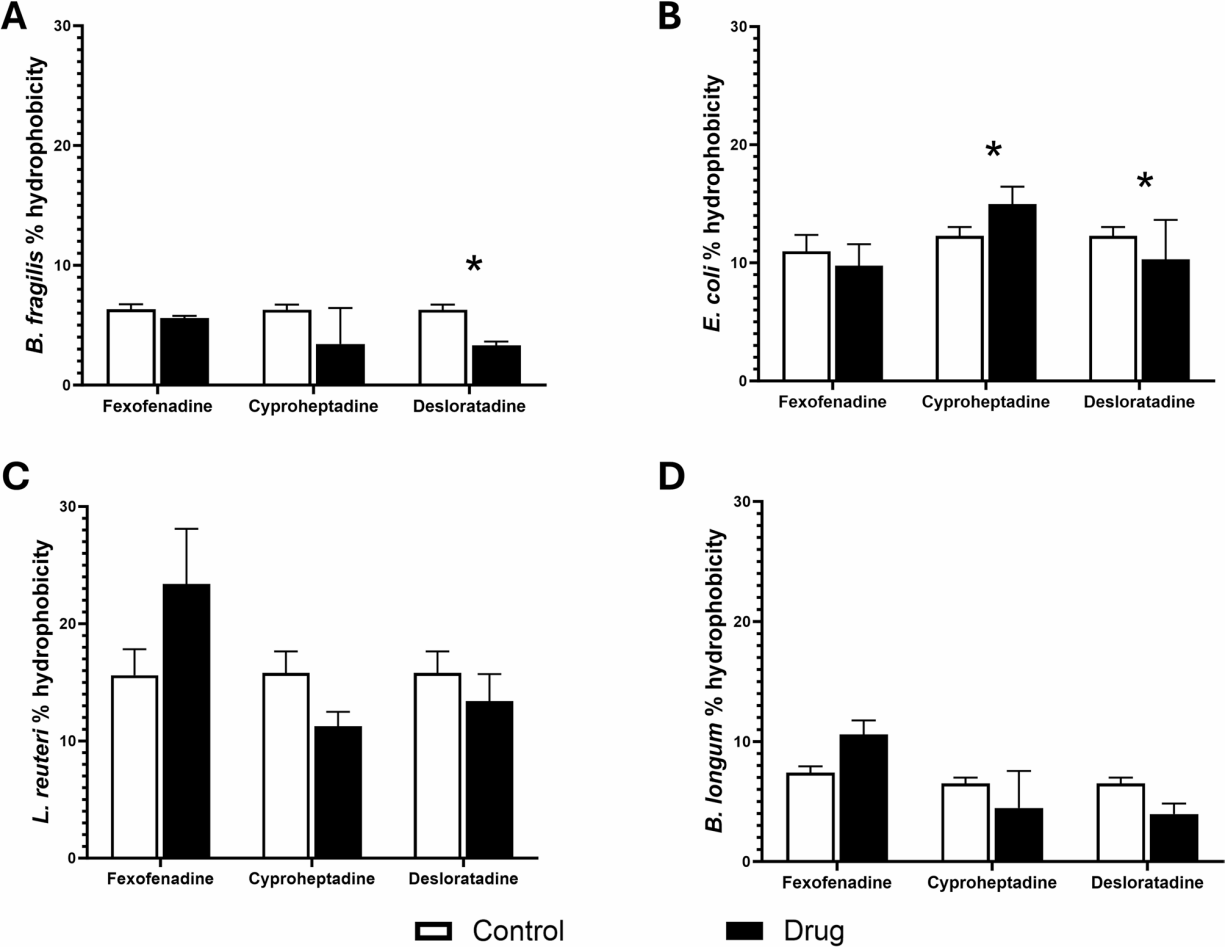
Hydrophobicity of bacteria in the presence of antihistamine drugs. Changes in percentage hydrophobicity were assessed for (**A**) *Bacteroides fragilis*, (**B**) *Escherichia coli*, (**C**) *Limosilactobacillus reuteri*, and (**D**) *Bifidobacterium longum* after incubation with antihistamines at 37 °C for 60 min. Data are presented as mean percentages from three independent experiments, with error bars representing standard error (SE). Tested antihistamines include fexofenadine HCl, cyproheptadine HCl, and desloratadine at intestinal concentrations of 37.165 µM, 4.1168 µM, and 5.3619 µM, respectively. The control group included bacteria with DMSO. Statistical analysis was conducted using multiple unpaired t-tests with Holm-Šídák correction for multiple comparisons. * Significant difference (*p* < 0.05)

The percentages of auto-aggregation (Figs. 2A-D) and cell surface hydrophobicity (Figs. 3A-D) varied among the tested antihistamines examined. Figure 2A showed that desloratadine significantly (*p* < 0.01) reduced the percentage of *B. fragilis* auto-aggregation, and Fig. 2D showed that cyproheptadine HCl did the same for *B. longum*. Furthermore, only Gram-negative bacteria, *B. fragilis* (Fig. 3A) and *E. coli (*Fig. 3B) had their percentage hydrophobicity reduced under the effect of desloratadine. Cyproheptadine HCl, on the other hand, caused an increase in *E. coli* percentage hydrophobicity (Fig. 3B).

### Antihistamines altered the bacterial ability to form biofilm in vitro

**Fig. 4:**
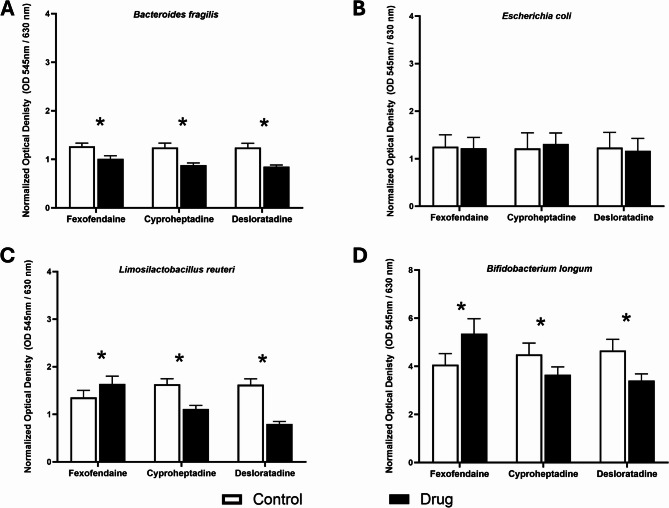
Biofilm formation ability of selected bacteria in the presence of antihistamine drugs. Changes in the biofilm formation index were measured for (**A**) *Bacteroides fragilis*, (**B**) *Escherichia coli*, (**C**) *Limosilactobacillus reuteri*, and (D) *Bifidobacterium longum* under the influence of antihistamines. Data are presented as mean percentages from three independent experiments, with error bars representing standard error (SE). Tested antihistamines include fexofenadine HCl, cyproheptadine HCl, and desloratadine at intestinal concentrations of 37.165 µM, 4.1168 µM, and 5.3619 µM, respectively. The control group consisted of bacteria cultured with DMSO. Statistical analysis was performed using multiple unpaired t-tests with Holm-Šídák correction for multiple comparisons. * Significant difference (*p* < 0.05)

The antihistamines exhibited an impact on the biofilm-forming ability of all bacterial species, except for *E. coli* (Figs. 4A-D). Cyproheptadine HCl and desloratadine were observed to have a diminishing effect on the biofilm formation of all bacteria. Fexofenadine HCl enhanced the capacity of Gram-positive bacteria for forming biofilm, as demonstrated in Fig. 4C and D. Conversely, it was observed to reduce the ability of *B. fragilis* to form a biofilm, as depicted in Fig. 4A.

### Effect of antihistamines on bacterial adherence to Caco-2/HT-29 co-culture cell lines

The intestinal concentration of all tested drugs showed no cytotoxic effect on co-culture cell lines except for desloratadine. Therefore 0.5x dilution of desloratadine was used (data not shown).

**Fig. 5:**
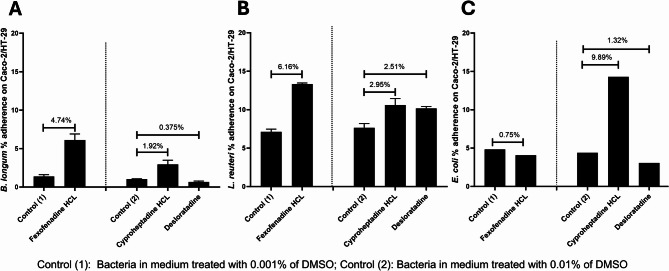
Bacterial adherence to Caco-2/HT-29 co-culture in the presence of antihistamine drugs. Changes in the percentage of adhered bacterial cells were evaluated for (**A**) *Bifidobacterium longum*, (**B**) *Limosilactobacillus reuteri*, and (**C**) *Escherichia coli* under the influence of antihistamines: fexofenadine HCl, cyproheptadine HCl, and desloratadine at intestinal concentrations of 37.165 µM, 4.1168 µM, and 2.68 µM (0.5x), respectively. Results are expressed as mean percentages from three independent experiments, with error bars indicating standard error (SE). The positive control included bacteria with DMSO at concentrations equivalent to those used for drug dissolution

*B. fragilis* adherence to the co-culture cell line was very weak and the antihistamine drugs did not increase their adherence (data not shown). The effect of antihistamine drugs on bacterial adherence was variable. Fexofenadine HCl increased the adherence of Gram-positive bacteria, as evidenced by Figs. 5A and B. Conversely, the adherence of *E. coli* was reduced, as demonstrated in Fig. 5C. The findings indicate that desloratadine exhibited a decrease in the adherence percentage of *B. longum* (Fig. 5A) and *E. coli* (Fig. 5C), while it demonstrated an increase in the adherence of *L. reuteri* to the cell lines co-culture in comparison to the control (Fig. 5B). Cyproheptadine HCl increased the adherence of all three bacterial species to the Caco-2/HT-29 co-culture.

### Imaging of Cyproheptadine HCl effect on adherence of *E. coli* to Caco-2/HT-29

**Fig. 6:**
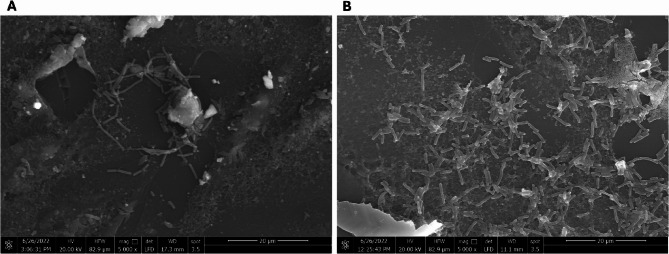
Scanning electron micrographs illustrating the effect of cyproheptadine HCl on *Escherichia coli* adherence to Caco-2/HT-29 co-culture. **A** Control showing bacterial adherence in the absence of the drug. **B** Drug-treated cells displaying altered bacterial adherence. Magnification: 5000x. The adherence assay was conducted in RPMI medium under 5% CO₂ at 37°C, and images were captured using a scanning electron microscope (Quanta 250 FEG, West Bengal, India)

Cyproheptadine HCl showed remarkable activity on the adherence of *E. coli* to the cell line co-culture. A scanning electron microscope was used to confirm its influence (Fig. 6).

### Effect of drugs on the production of SCFAs and lactic acid

The standard solution’s reference chromatograms showed that SCFAs: lactic, acetic, and propionic acids propionic were found at distinct retention times: 13.53, 15.65 and 17.42 min, respectively. In both bacterial species (*B. longum* and *L. reuteri*), the concentrations of all three SCFAs were higher in the presence of fexofenadine HCl compared to the control group (Table 1). The concentration of butyric acid was below the detection limit all drugs treated samples at retention time 21.5 min. All SCFA concentrations are reported as mean ± SD, along with their coefficients of variation. Statistical analysis showed that only the increase in acetic acid was statistically significant (*p* < 0.05).

**Table 1:** Concentration of SCFAs and Lactic Acid Produced by *Bifidobacterium longum* and *Limosilactobacillus reuteri* in Response to Fexofenadine HCl, Determined via HPLC Analysis

**Compound** ** (RT in mins)**	***B. longum*** ** Untreated (ppm)**	***B. longum*** ** Treated (ppm)**	***L. reuteri*** ** Untreated (ppm)**	***L. reuteri*** ** Treated (ppm)**
Lactic acid (13.53)	109.74 ± 20.22 (18.4 %)	167.00 ± 44.40 (26.6 %)	896.81 ± 147.99 (16.5 %)	1141.26 ± 20.82 (1.8 %)
Acetic acid (15.65)	2526.94 ± 186.76^*^ (7.39 %)	3317.20 ± 147.63^*^ (4.45 %)	2802.80 ± 186.55^*^ (6.7 %)	3513.74 ± 246.62^*^ (7.0 %)
Propionic acid (17.42)	279.23 ± 42.46 (15.2 %)	380.97 ± 6.74 (1.77 %)	305.68 ± 53.62 (17.5 %)	366.00 ± 1.54 (0.4 %)
Butyric acid was below the detection limit in all samples				

The values are shown as the mean ± SD (coefficient of variation)

*Significant difference (*p* < 0.05)

## Discussion

In this study, we assessed the impact of estimated intestinal concentrations of commonly used long-term antihistamines, such as cyproheptadine HCl, desloratadine, and fexofenadine HCl, on the growth of selected bacterial species, their ability to form biofilms and their adherence to cell lines.

Histamine is a human neurotransmitter that modulates inflammatory reactions, the immune response, and the regulation of gastrointestinal, cardiovascular, and circulatory functions. It acts through four types of histamine receptors: H_1_R, H_2_R, H_3_R, and H_4_R. Histamine has become the focus of research due to its role as a signaling molecule in bacteria-host interactions. Histamine sensing by bacteria can regulate the expression of some virulence genes [38]. Detection of histamine by bacteria can serve several purposes: (i) indicating the presence of a compound of metabolic value or toxicity, or (ii) informing the bacterium about its current environmental niche. A wide range of Gram-positive and Gram-negative bacteria have been shown to synthesize histamine [38] while others exhibit metabolizing activity for histamine [39].

Our data revealed that cyproheptadine HCl significantly reduced the viable bacterial count by 1 to 2 logs, unlike fexofenadine HCl aligning with findings by El-Nakeeb et al. [40]. Similarly, another study showed that cyproheptadine HCl exhibited high antimicrobial and antibiofilm activity against *Klebsiella pneumonia* compared to other tested antihistamines [41]. Desloratadine, like cyproheptadine HCl, displayed antimicrobial activity against all bacteria. Our findings are consistent with Eduvirgem et al. [42], who reported that desloratadine has inhibitory and anti-biofilm effects. However, Maier et al. [19]reported that fexofenadine HCl exhibits antimicrobial activity against *E. coli*, while cyproheptadine HCl has a similar effect on *Bacteroides uniformis*. Our findings partially align with these results, as we observed that cyproheptadine HCl inhibited the growth of *B. fragilis*, whereas fexofenadine HCl enhanced the growth of *E. coli*.

To explore the reasons behind the diverse antimicrobial effects, it was suggested that some antihistamines might be adsorbed onto bacterial cell surfaces, potentially enhancing impact on cell membranes. Surface activity is said to increase with hydrophobicity. The degree of adsorption onto membranes due to surface activity has been linked to detrimental effects [43]. Cyproheptadine HCl, with a tertiary amino group and a bulky lipophilic aromatic moiety as its primary structural components, possesses certain surfactant-like properties [36]. Amphipathic compounds’ surface activity may alter the function and permeability of biological membranes [44]. Chlorine in desloratadine increases its surface activity [45]. Fexofenadine HCl, however, has significant hydrophilic characteristics due to a hydroxyl group substituent, which significantly reduces its surface activity and potential effects on membranes [45]. Chemical compounds with piperidine rings have been reported to have antimicrobial activity against several infections [46]. Despite this, fexofenadine HCl —a piperidine-based antihistamine—significantly increased the growth of *B. fragilis*,* E. coli*, and *L. reuteri* while notably reducing the abundance of *B. longum.* This could be attributed to the hydrophilic properties of fexofenadine HCl imparted by the hydroxyl group substituent, resulting in reduced surface activity and its potential effects on membranes, as well as the ability of bacteria to utilize fexofenadine HCl [40, 47].

Bacterial adhesion to different surfaces is a complex process involving contact between bacterial membranes and interacting surfaces. Factors contributing to bacterial adherence to different surfaces and, consequently, contribute to the development of various diseases, include bacterial adhesive structures, secretory activity, and matrix composition [48]. Specific and nonspecific binding are two distinct strategies causing bacterial adhesion. Electrostatic or hydrophobic interactions play a major role in nonspecific binding and significantly affect adhesion strength [49, 50]. Auto-aggregation and CSH assays were conducted to indirectly evaluate the effect of the drugs on bacterial adherence, followed by studies on biofilm formation and bacterial adherence to cell line co-cultures (Caco-2/HT-29). We observed that changes in hydrophobicity of Gram-negative bacteria were associated with a change in their adherence to cell lines. Increased hydrophobicity in *E. coli* with cyproheptadine HCl was linked to increased adherence to the cell line, while reduced hydrophobicity of *E. coli* with desloratadine was associated with decreased adherence. Conversely, auto-aggregation results were more closely linked to biofilm formation than adherence to cell lines.

A wide range of Gram-positive and Gram-negative bacteria possess histidine decarboxylase (HDC) encoding genes and can synthesize histamine [51]. Histamine acts as a bacterial signaling molecule, influencing chemotaxis and virulence factor expression by binding to specific bacterial receptors [11, 52]. The gut commensal *L. reuteri* produces histamine, which can modulate immune responses through H2 receptors (Shelby et al., 2022). This underscores the complex interplay between bacteria, histamine, and host factors in the gut environment. Given the pivotal role of histamine in bacterial pathogenesis, we hypothesize that antihistamine drugs may exert antimicrobial and anti-adherence effects by interfering with bacterial histamine signaling or binding to histamine binding receptors. The differential impacts of antihistamines on gut microbiota could be attributed to their varying histamine receptor selectivity. First-generation agents like cyproheptadine broadly target histamine H1 receptors alongside other receptors [53], while second-generation antihistamines generally demonstrate greater H1 receptor specificity [52]. Notably, within this newer class, desloratadine displays reduced selectivity compared to fexofenadine due to its affinity for additional receptor subtypes [54]. These differential receptor binding profiles may contribute to the observed differences in the impact of these drugs on gut microbiota.

The composition of the gut microbiota in individuals with allergic conditions remains inconsistent [55]. However, increased Bacteroides and Escherichia have been linked to non-allergic phenotypes [56]. Our findings suggest that fexofenadine HCl promotes a gut microbiota profile associated with improved allergic outcomes. Fexofenadine HCl significantly enhanced the growth and colonization of beneficial bacteria, such as *Lactobacillus reuteri* and *Bifidobacterium longum*. These organisms are known to exert anti-inflammatory effects and alleviate allergic symptoms [57–60]. Moreover, fexofenadine HCl stimulated the production of anti-inflammatory short-chain fatty acids (SCFAs) by these bacteria, further supporting its potential role in allergy management [61]. The short-chain fatty acids acetate, lactate, and propionate have demonstrated anti-allergic effects in various studies, primarily through their immunomodulatory properties [62–64]. In contrast, cyproheptadine HCl and desloratadine had detrimental effects on the gut microbiota, reducing the abundance of beneficial bacteria while promoting their adherence to the gut epithelium.

One limitation of this study is that the evaluated antihistamines may be metabolized in the human body prior to reaching the gut. Fexofenadine is primarily excreted unmodified, whereas desloratadine and cyproheptadine are subject to hepatic metabolism, which may modify their interactions with gut bacteria [65]. Future studies should explore the impact of these drugs metabolites in in vivo conditions. While the current study provides insights into the in vitro effects, it does not fully explore the in vivo implications or the underlying molecular bases of microbial mechanisms. Nevertheless, the findings offer a foundation for future investigations. 

## Conclusion

This study highlights the differential effects of antihistamine drugs on gut microbiota by assessing their impact on the growth, biofilm formation, and adherence of selected gut bacterial species at intestinal concentrations. Our findings highlight the differential effects of these drugs, with fexofenadine HCl found to promote the growth and colonization of probiotic bacteria such as *Limosilactobacillus reuteri* and *Bifidobacterium longum*, enhancing their biofilm formation, adherence to cell lines, and production of SCFA, which is known for its antiallergic properties. In contrast, cyproheptadine HCl and desloratadine reduced the growth of all tested bacteria and decreased their biofilm formation and adherence capabilities, suggesting their potential antimicrobial effects. These findings indicate that fexofenadine HCl could support probiotic health and offer additional antiallergic benefits, while cyproheptadine HCl and desloratadine might be repurposed for antimicrobial applications. Understanding the specific interactions between these antihistamines and gut microbiota provides valuable insights into their broader implications for gut health and disease prevention. Future research should further explore the mechanisms behind these interactions and their potential therapeutic applications.

## Data Availability

All data supporting the findings of this study are available within the paper and upon request from the corresponding author.
